# Availability and use of magnesium sulphate at health care facilities in two selected districts of North Karnataka, India

**DOI:** 10.1186/s12978-018-0531-6

**Published:** 2018-06-22

**Authors:** Geetanjali Katageri, Umesh Charantimath, Anjali Joshi, Marianne Vidler, Umesh Ramadurg, Sumedha Sharma, Sheshidhar Bannale, Beth A. Payne, Sangamesh Rakaraddi, Chandrashekhar Karadiguddi, Geetanjali Mungarwadi, Avinash Kavi, Diane Sawchuck, Richard Derman, Shivaprasad Goudar, Ashalata Mallapur, Mrutyunjaya Bellad, Laura A. Magee, Rahat Qureshi, Peter von Dadelszen, the Community Level Interventions for Pre-eclampsia (CLIP) India Feasibility Working Group

**Affiliations:** 1 Department of Obstetrics and Gynaecology, S Nijalingappa Medical College, Bagalkot, Karnataka India; 2 KLE Academy of Higher Education and Research’s J N Medical College, Belagavi, Karnataka India; 3 Department of Obstetrics and Gynaecology, University of British Columbia, Vancouver, BC Canada; 4 Department of Community Medicine, S Nijalingappa Medical College, Bagalkot, Karnataka India; 5 Department of Pharmacology, S Nijalingappa Medical College, Bagalkot, Karnataka India; 6 Department of Anatomy, S Nijalingappa Medical College, Bagalkot, Karnataka India; 7 School of Life Course Sciences, Faculty of Life Sciences and Medicine, King’s College London, London, UK; 8 Global Affairs, Thomas Jefferson University, Philadelphia, USA; 9 Division of Women and Child Health, Aga Khan University, Karachi, Sindh Pakistan; 10 Department of Research, Vancouver Island Health Authority, British Columbia, Canada

**Keywords:** Magnesium Sulphate, Availability, Health care facilities, Pre-eclampsia, Eclampsia, Karnataka, India

## Abstract

**Background:**

Pre-eclampsia and eclampsia are major causes of maternal morbidity and mortality. Magnesium sulphate is accepted as the anticonvulsant of choice in these conditions and is present on the WHO essential medicines list and the Indian National List of Essential Medicines, 2015. Despite this, magnesium sulphate is not widely used in India for pre-eclampsia and eclampsia. In addition to other factors, lack of availability may be a reason for sub-optimal usage. This study was undertaken to assess the availability and use of magnesium sulphate at public and private health care facilities in two districts of North Karnataka, India.

**Methods:**

A facility assessment survey was undertaken as part of the Community Level Interventions for Pre-eclampsia (CLIP) Feasibility Study which was undertaken prior to the CLIP Trials (NCT01911494). This study was undertaken in 12 areas of Belagavi and Bagalkote districts of North Karnataka, India and included a survey of 88 facilities. Data were collected in all facilities by interviewing the health care providers and analysed using Excel.

**Results:**

Of the 88 facilities, 28 were public, and 60 were private. In the public facilities, magnesium sulphate was available in six out of 10 Primary Health Centres (60%), in all eight *taluka* (sub-district) hospitals (100%), five of eight community health centres (63%) and both district hospitals (100%). Fifty-five of 60 private facilities (92%) reported availability of magnesium sulphate.

Stock outs were reported in six facilities in the preceding six months – five public and one private. Twenty-five percent weight/volume and 50% weight/volume concentration formulations were available variably across the public and private facilities. Sixty-eight facilities (77%) used the drug for severe pre-eclampsia and 12 facilities (13.6%) did not use the drug even for eclampsia. Varied dosing schedules were reported from facility to facility.

**Conclusions:**

Poor availability of magnesium sulphate was identified in many facilities, and stock outs in some. Individual differences in usage were identified. Ensuring a reliable supply of magnesium sulphate, standard formulations and recommendations of dosage schedules and training may help improve use; and decrease morbidity and mortality due to pre-eclampsia/ eclampsia.

**Trial registration:**

The CLIP trial was registered with ClinicalTrials.gov (NCT01911494).

**Electronic supplementary material:**

The online version of this article (10.1186/s12978-018-0531-6) contains supplementary material, which is available to authorized users.

## Background

Pre-eclampsia and eclampsia are a leading cause of maternal mortality, contributing to 14% of maternal deaths worldwide [1]. Every day approximately 830 women die as a result of pregnancy complications all over the world and 99% of these deaths occur in developing countries [2]. In 2015, globally there were 303,000 maternal deaths; and 45,000 (about 15%) of these were in India [3]. India has fallen short of achieving the Millennium Development Goal of reducing the national maternal mortality ratio (MMR) to 109 by 2015. Despite a substantial reduction in maternal deaths, the MMR of India stood at 174 per 100,000 livebirths in 2015 [3]. It is now necessary to focus on the newly adopted Sustainable Development Goals targeted to reduce the global MMR to 70.

Interventions targeting maternal deaths need to address pre-eclampsia and eclampsia as it is the second leading direct cause of maternal mortality, second only to haemorrhage [1]. Also, these conditions can result in major morbidity and residual complications which affect the quality of life of the woman and her family and significant economic burden.

Magnesium sulphate is the drug of choice for the prevention and treatment for the seizures of eclampsia [4]. The Magpie trial established that women receiving magnesium sulphate versus placebo were 58% less likely to have convulsions and it also led to a decreased risk of placental abruption. Follow-up studies of the participants of the Magpie trial and their babies showed no long-term harm or benefit to either [5–7]. A systematic review of the Cochrane database in 2010 found that magnesium sulphate is better than phenytoin and nimodepine in preventing convulsions and results in a non-significant decrease in maternal deaths [8]. Studies have also proven that it prevents convulsions better when compared to the other treatment regimens for eclampsia like the lytic cocktail regimen and diazepam [9, 10].

The World Health Organization’s (WHO) list of essential medicines and the Indian National List of Essential Medicines (NLEM) 2015 identify magnesium sulphate as an essential commodity for maternal health [11, 12]. It is interesting to note that the NLEM lists it under S and T categories which means that it should be available at secondary and tertiary facilities, when in contrast, the Indian Public Health Standards (IPHS) Revised Guidelines 2012, specifically the IPHS guidelines for primary health centres (PHCs), state that magnesium sulphate should be present in the labour room in the PHCs [13]. The antidote to magnesium sulphate, calcium gluconate is listed on the WHO list of essential medicines and also features on the NLEM 2015 under categories P, S and T, implying that it should be present at the primary, secondary and tertiary levels.

The guidelines for skilled birth attendants in India recommend that magnesium sulphate be administered in cases of severe pre-eclampsia and eclampsia prior to referral [14]. These guidelines are meant to guide the functioning of Staff Nurses (providing services at the primary health centres and higher facilities), Auxiliary Nurse Midwives (ANMs; providing services at the subcenters) and Lady Health Visitors (LHVs) who are in a supervisory capacity for the ANMs. The guidelines also recommend that magnesium sulphate be present in the kit for home delivery and specify that the skilled birth attendants are permitted to use magnesium sulphate for both eclampsia and severe pre-eclampsia, and that arrangements for immediate referral should then be made [14]. Providing the loading dose of magnesium sulphate will stabilize a woman for safe transport to a facility, which is better equipped to handle these complications. Despite all the recommendations, use of magnesium sulphate is not universal [15].

There may be many reasons for the sub-optimal use of magnesium sulphate, such as a lack of knowledge, lack of familiarity with the drug, the large volume of the injection, apprehension of complications, widely varied doses and dosage schedules that may be confusing; among others [15]. Though there are standard regimens described in obstetric textbooks, there are concerns that the dose may be too large for the smaller built Indian woman. Hence several modified regimens are followed in practice [16–19]. In addition to this, magnesium sulphate may not be readily available in all settings [15].

We undertook an assessment of health facilities in two districts of North Karnataka to note how they were equipped in handling obstetric and neonatal care. In this study, we report the availability and use of magnesium sulphate at the facilities assessed.

## Methods

This study was carried out in Belagavi and Bagalkote districts of North Karnataka. Karnataka is a south Indian state with a MMR of 133/100,000 livebirths in 2011-2013 as per the Sample Registration System. There are vast differences in health infrastructure and service delivery in the districts of northern and southern Karnataka [20]. The CLIP Feasibility Study was undertaken in 12 areas of Belagavi and Bagalkote districts (six in each district) [21]. The two adjoining districts are quite dissimilar, with poorer health indices in Bagalkote district [20]. Before the CLIP Trial (NCT01911494), the 12-month Feasibility Study was undertaken to assess the context, and identify any potential barriers and facilitators to the implementation of CLIP.

As a part of the CLIP Feasibility Study, facility assessment was undertaken to determine the capacity of facilities in the study area in the provision of maternal and newborn health services, with a focus on care of women with pre-eclampsia and eclampsia. Medical officers, in charge of provision of care at primary health centres (PHC), were asked to identify and list the health care facilities that women in their area frequent. In total, 88 facilities were identified. The survey of the facilities was undertaken between April and August 2013.

The survey was carried out by trained medical professionals from the research team from the same region, who underwent training for a day in the approach necessary for data collection. Those conducting assessments were predominantly clinicians; this provided important background knowledge for successful completion of the forms. The primary obstetric care provider at each facility was approached for a one-on-one interview to provide necessary information, with some fields being completed with inputs from pediatric care providers, laboratory technicians and review of institutional records. Consent was obtained from the respondents before proceeding.

A pre-structured questionnaire was used to document the responses received. The questionnaire recorded preliminary data about the name of the facility, location, person responding to the questionnaire, contact numbers, type of facility, usual place of referral, catchment area and population served. It also included the number of beds, availability of intensive care units, a comprehensive description of the available personnel, equipment, drugs and services, costs, transportation to and from the facility, access to blood transfusion, and the volume of patients. Questions pertaining to additional training in maternal and neonatal care were included in the format, as were those related to existence and use of facility guidelines for the management of hypertensive disorders of pregnancy. Questions regarding hypertension in pregnancy, criteria used for diagnosis of mild and severe pre-eclampsia, antihypertensive treatment, and use of magnesium sulphate were also asked. The majority of the questions in the survey tool were close-ended though a few questions requested detailed answering. Data were directly recorded on to hard copies of the questionnaire at the time of the interview. The questionnaire used in this study has been added as an appendix to this paper (Additional file 1).

All data were keyed into a locally developed Access database. The data were cleaned prior to analysis. Frequencies were run on all quantitative fields providing totals, means and standard deviations.

This study was approved by ethics review committees at the University of British Columbia, Vancouver Canada (H12-00132) and KLE University, Belgaum (Ref No: MDC/IECHSR/2013-14/A-28) India.

## Results

Of the 88 facilities, 28 were public and 60 were private. Table 1 provides a description of the facilities surveyed.

**Table 1:** Facilities assessed, the availability of magnesium sulphate at assessment and stock outs in preceding 6 months

Type of facility		Belagavi district			Bagalkote District			Total		
		N	A	S	N	A	S	N	A	S
Public facilities	Primary Health Centre	9	5	2	1	1	0	10	6	2
	Community Health Centre	4	3	0	4	2	2	8	5	2
	Taluka Hospital	4	4	0	4	4	1	8	8	1
	District Hospital	1	1	0	1	1	0	2	2	0
Private facilities		39	35	0	21	20	1	60	55	1
All facilities		57	48	2	31	28	4	88	76	6

*N* Number assessed

*A* Magnesium sulphate available during survey

*S* Stock outs of magnesium sulphate in preceding six moths

The facilities assessed included 8 Community Health Centres (CHC) out of the total 24 in the two districts (33%) as reported by the Health Management Information System of the Ministry of health and Family welfare; 8 *taluka* hospitals out of the total 14 (57%), and both the district hospitals (100%). The total number of private obstetric facilities in the two districts could not be ascertained to determine the proportion assessed.

All health care facilities with magnesium sulphate at the time of assessment also reported access to the drug in the labour room. Data regarding stock outs in the preceding 6 months were collected. While the majority of the healthcare facilities did not report any stock outs, five public facilities did report stock outs at the time of assessment, one reporting multiple instances in the preceding 6 months. One private facility reported a stock out. One CHC and one private hospital reported having no availability of magnesium sulphate at any time and hence there was no response to whether stock outs occurred. One PHC medical officer reported that they did not wait for replenishment of stock through the government supply since it was not streamlined, and they bought the necessary supply through funds allocated to the PHC. Table 1 shows the different categories of health facilities surveyed, availability of magnesium sulphate at the time of assessment and stock outs in the preceding 6 months.

The availability of other essential obstetric medications was ascertained. There were differences in availability, with some facilities reporting availability of one type of a drug in a category versus another (for example betamethasone/dexamethasone, different antihypertensives etc.) and some reporting availability of all the drugs. All facilities reported the availability of uterotonics, oxytocin in particular.

Regarding the formulation of magnesium sulphate available, responses were collected from 29 facilities with magnesium sulphate at the time of assessment. Of these, 22 (6 public and 16 private) facilities reported carrying a concentration of 50% weight /volume (*w*/*v*), three (one public and 2 private) reported having 25% w/v and four (one public and 3 private) reported having both strengths available. All ampoules were of 2 ml.

Information on practices related to magnesium sulphate usage was collected, including the use of magnesium sulphate in the management of severe pre-eclampsia (Table 2). Health care professionals from 68 facilities responded that they did use it for management of severe pre-eclampsia, and notably this included five PHCs. Thirty-two providers reported differential dosing schedules for severe pre-eclampsia as opposed to eclampsia with differences in both the route and the dosage of magnesium sulphate used. Seventeen of the 68 providers reported that they did not provide a maintenance dose in cases of severe pre-eclampsia. One private practitioner reported maintenance therapy with phenytoin after the loading dose of magnesium sulphate for pre-eclampsia. All the PHCs and two private hospitals using magnesium sulphate reported immediate referral of patients with severe pre-eclampsia and eclampsia, without waiting for the maintenance dose.

**Table 2:** Facilities using magnesium sulphate for management of severe pre-eclampsia

Use of MgSO_4_ for severe pre-eclampsia		
Yes	Public	17
	Private	51
	Total	68
No	Public	4
	Private	4
	Total	8
Never used MgSO_4_ for even eclampsia	Public	7
	Private	5
	Total	12

When asked about alternative treatment strategies in eclampsia in place of magnesium sulphate amongst those using the drug, the majority of the providers mentioned use of injection diazepam or phenytoin, but only in cases where magnesium sulphate failed. Only one provider mentioned that maintenance treatment was routinely provided with phenytoin.

A notable observation was that 12 providers did not use magnesium sulphate even for eclampsia. This included 4 PHCs, 3 CHCs and 5 private facilities. Three of these 12 facilities did report availability of magnesium sulphate at the time of assessment. Four of the 12 providers mentioned the use of injectable diazepam for eclampsia.

Varied dosage schedules were reported, with 25 (3 public and 22 private) of 68 providers reporting using standard Pritchard or Zuspan regimens for severe pre-eclampsia, and 44 (13 public and 31 private) of 76 reporting use of standard Pritchard or Zuspan regimens for eclampsia. The dosages reported were very varied, with most providers who were not using standard regimens resorted to lower doses, some up to half of the Pritchard and Zuspan regimens. One obstetrician at a private facility reported the use of 2 g intramuscularly (IM) as the loading dose and 2 g IM twice a day as the maintenance dose for eclampsia. There were also some unusual responses like 100 mg intravenously (IV) every 8 h; and 25% IM. The respondents in these cases were not obstetricians by qualification but were still the primary obstetric providers in that facility. Interestingly, 10 providers said they determine the loading and maintenance doses based on the patient’s condition.

The availability of calcium gluconate, the antidote to magnesium sulphate was asked for. Calcium gluconate was not available in 12 facilities, including 8 which reported availability of magnesium sulphate (Table 3).

**Table 3:** Non-availability of calcium gluconate by type of facility

Type of facility	Calcium gluconate not available	Calcium gluconate not available but magnesium sulphate available
PHC	2	0
Taluka Hospital	1	1
CHC	2	1
Private	7	6

## Discussion

Despite the well-established superiority of magnesium sulphate in the management of severe pre-eclampsia and eclampsia, it continues to be used sub-optimally. In our study, non-availability of magnesium sulphate was identified at several facilities. In addition, stock outs were experienced at five public facilities and one private secondary facility in the preceding 6 months but in none of the tertiary health facilities. Calcium gluconate was available in 86% of the facilities assessed but 8 facilities with availability of magnesium sulphate did not have calcium gluconate.

It was found that the health facilities had differing strengths of magnesium sulphate available, some reporting both 25% *w*/*v* and 50% w/v. The facilities reported varying dosage schedules, some of which may not have the required stabilizing action and may not optimally benefit the patient.

The availability of magnesium sulphate seemed to be similar across the two districts in this study. However, there were more stock outs in the preceding 6 months in Bagalkote when compared to Belagavi. Also, the stock outs were in the secondary level facilities in Bagalkote as opposed to primary health centres in Belagavi. Availability and use of magnesium sulphate across all levels of healthcare is important for ensuring favourable outcomes.

The fact that other essential medications for obstetric health were available at the facility and only magnesium sulphate seemed to be missing, points to the fact that the supply chain is generally functioning. Due importance is not given to make magnesium sulphate available. The providers at the public facilities do have funds to procure the drug through other suppliers but have to maintain a watch on the stock to see that the supply is uninterrupted.

The facilities assessed in this study were the ones most often used for delivery and it would be expected that these health facilities would have better logistics than those not providing regular obstetric services. It is probable that this assessment does not represent all facilities in the region, and that others may have poorer availability and use of magnesium sulphate. The strength of this study is that researchers were trained medical professionals and hence had a better contextualization of the study. A limitation of this study is that the health care providers self-reported the availability and use of magnesium sulphate in the facilities they served. It was not physically ascertained as to how many ampoules were present, whether the ampoules were within the date of expiry, and whether magnesium sulphate was used in the manner reported. Though the researchers encouraged practitioners to report actual practices, the fact that the researchers were fellow clinicians may have induced a bias to report favourably.

A study assessing 279 health care facilities in eight districts of northern Karnataka in 2010 revealed that magnesium sulphate was available in only 18% of PHCs, 48% of higher public facilities and 70% of private facilities [22]. In comparison, the present study found that 60% of PHCs, 72% of higher public facilities and 92% private facilities reported availability of magnesium sulphate at the time of assessment. This difference may be because we selectively sampled facilities providing regular obstetric services.

A study from Maharashtra state, India assessing 44 secondary and tertiary public health facilities in 2009-2010 found that 61% of facilities had no stock of magnesium sulphate, with the stock-out position ranging from 3 months to 3 years. The researchers ascertained whether they had the minimum of 50 ampoules recommended in the guidelines for CHCs and found that 20% facilities had less than the minimum recommended and also that 11.4% of the assessed facilities did not have enough ampoules to even provide the first dose to a single woman [23]. The stock outs in the present study were much fewer, with only five of 28 public facilities (18%) reporting stock outs in the 6 months before the survey, again perhaps due to the convenience sampling used in this study. The quantity of drug available was not assessed in this study.

Another study done in Nagpur, Maharashtra concluded that though senior gynaecologists favoured the use of magnesium sulphate especially prior to referral, its use was limited due to lack of institutional policies on dosing, timing, indications and also due to limited availability, especially in tertiary care centres [24]. In the present study too, it was found that health care providers did not have uniform policies on indications and dosing, even when the drug was available.

A 2010 WHO study that assessed emergency obstetric care across six developing countries, including India, found that only 53% of facilities with basic emergency obstetric care (BEmOC) and 86% of facilities with comprehensive emergency obstetric care (CEmOC) had IM/IV anticonvulsants [25]. The study also indicated that public facilities were unable to provide emergency obstetric care due to lack of good management systems to ensure continuous availability of drugs and supplies and emphasized the importance of strengthening the chain for procurement and distribution of basic drugs and equipment; and to improve skills of the providers to ensure at least coverage of BEmOC [25]. In the present study, in addition to the issues about availability of magnesium sulphate, we found that providers at 12 facilities did not administer magnesium sulphate even for eclampsia despite three of these facilities reporting availability. This emphasizes the need for knowledge and skill enhancement activities to improve usage practices in addition to improving the supply chain.

Even though the guidelines for skilled birth attendants [14] recommend that the ANM should administer magnesium sulphate for severe pre-eclampsia prior to referral, the medical officers (who are qualified physicians) of five of the PHCs stated that they did not use magnesium sulphate for severe pre-eclampsia and despite availability of magnesium sulphate, usage for even eclampsia was not universal. Familiarising health care professionals with treatment recommendations, and building their capacity and confidence could go a long way in optimizing the administration of magnesium sulphate.

There are standard regimens for the use of magnesium sulphate, Pritchard, Zuspan and Sibai; as well as, many other dosage schedules [16–19]. A systematic review of the studies evaluating these regimens in low- and middle-income countries was unable to establish the superiority of any regimen and the lowest effective dose is not agreed upon [26]. It was found in four studies that the administration of the loading dose only was as effective as loading plus maintenance [26]. The varied dosage schedules, however, confuse practitioners who may not be familiar with magnesium sulphate. The strength of the drug formulation also varies and achieving the correct dilution for IV administration may be prone to error and confusion [27]. In the present study, we did find that the health facilities had differing strengths available and used many differing dosing schedules. It is important that there be a standard formulation and requirement for adherence to standard dosing schedules to increase appropriate use and optimize action.

Magnesium sulphate toxicity occurs very infrequently if guidelines are followed during administration. In the event that it does occur, serious complications can usually be averted by skipping the next dose. However, in rare instances, administration of calcium gluconate is required and may be lifesaving [28]. Hence, care should be taken to ensure availability and train the providers in appropriate use. A study in Lucknow, India in 2014 found that only 33.3% *Bal Mahila Chikitsalayas* (health care facilities for women and children) which serve as first referral units had availability of calcium gluconate [29]. In our study, availability was found to be better with 86% facilities reporting the drug at the time of assessment.

The Indian guidelines are conflicting, with the NLEM recommending magnesium sulphate availability in the secondary and tertiary facilities, the IPHS guidelines for PHCs recommending availability in the labour room of the PHCs; and the guidelines for skilled birth attendants recommending that it be available at the sub-center and even in the home delivery kits [12–14]. Conflicting recommendations of this nature need to be rectified to ensure enhanced usage.

The first step to encourage enhanced magnesium sulphate use is that it should be available at all health care facilities providing for obstetric patients, without any stock-outs. It is also essential to increase the providers’ comfort and confidence in using this drug. All levels of obstetric care providers need to be re-trained periodically in the benefits and use of magnesium sulphate.

Observing the appropriate use of magnesium sulphate by senior obstetric providers may enhance uptake by the other health care providers like resident doctors, ANMs and staff nurses. However, they need to be exposed to the use of this drug in a consistent manner. Standardizing the strength and dosing schedules is essential; and guidelines for referral should be developed and enforced. Identification of non-compliant facilities could be done by tracking referrals. Ascertaining the cause for deviation with corrective action and refresher training could be a solution to increase appropriate use. Cases of eclampsia could also serve as triggers for evaluating the delays and deficiencies in the health system and identifying and implementing potential remedial action.

Future research could focus on the reasons for hesitancy for use of magnesium sulphate by obstetric providers so as to inform the health system administrators about the steps necessary to increase usage.

## Conclusion

This study found that there were deficiencies in the availability of magnesium sulphate in health care facilities that routinely cared for obstetric patients in north Karnataka. The public health facilities faced stock outs though they could procure the drug through funds allocated to them. This shows a poor chain of coordination between the suppliers, distributors and the users.

Addressing contradictions in the Indian national guidelines regarding place of availability, indications for use and cadre of health care professionals permitted to use magnesium sulphate is of prime importance.

The indications for which magnesium sulphate was used and the strength of drug available varied across facilities of the same type. Standardization of the drug formulation and familiarization with guidelines is important to ensure optimal use of magnesium sulphate. In addition, identification of the factors preventing use; and trainings and refresher trainings to address these issues would increase appropriate usage of magnesium sulphate.

Despite a major reduction in the maternal mortality, the Millennium Development Goals of 2015 were not met in India; nevertheless, positive action in this regard could bring us closer to attaining the Sustainable Development Goals by 2030.

## Additional file

Additional file 1: Questionnaire used in this study. (PDF 607 kb)

## Abbreviations

ANM: Auxillary nurse midwife
BEmOC: Basic emergency obstetric care
CEmOC: Comprehensive emergency obstetric care
CHC: Community Health Centre
CLIP: Community level interventions for pre-eclampsia
IM: Intramuscular
IPHS: Indian Public Health Standards
IV: Intravenous
LHV: Lady health visitor
MMR: Maternal mortality ratio
NLEM: National list of essential medicines
PHC: Primary health centre
SN: Staff nurse
*w*/*v*: Weight/volume
WHO: World Health Organization
