# Supplementary material for: Availability and use of magnesium sulphate at health care facilities in two selected districts of North Karnataka, India
Source: Reprod Health. 2018 Jun 22;15(Suppl 1):91. doi: 10.1186/s12978-018-0531-6 (PMC6020005; doi:10.1186/s12978-018-0531-6)
Supplement: Supplementary file 1 — Questionnaire used in this study. (PDF 607 kb) [file 12978_2018_531_MOESM1_ESM.pdf]

HEALTH FACILITY ASSESSMENT QUESTIONNAIRE : CLIP TRIAL, INDIA

The purpose of the health facility assessment questionnaire is to determine the capacity of this facility in the provision of maternal-newborn care services, with a focus on care of women with Pre-eclampsia and Eclampsia.

Note: Unless otherwise indicated, entire information obtained should enter directly in the appropriate column/place by using the codes given separately, Please use [999] for "No answer/Don't know"; [888] for "Not applicable" [1] for "Yes" and [2] for "No".

Date of Assessment ______________________

Name of Assessor __________________________

Respondent Name ________________________

Respondent Position / designation_________________

SECTION 1: IDENTIFICATION [*Follow area/facility code list*]

- District _________________
- Type of Facility
    - Primary health Center
    - CHC/FRU
    - Taluka Hospital
    - Tertiary Hospital/district hospital
    - Private Hospital/private nursing home
- Facility ID number _______________
- Name of the Facility _____________
- Facility address with landmarks ____________________________________ ______________________________________ (Include PIN code/Zip code)
- Facility GPS Coordinate/geo-tag _______________
- Higher level referral to____________________ Distance ___________

1.8 Telephone with STD code :

Landline _____________

Mobile +91_______________

SECTION 2 :GENERAL INFORMATION

2.1 Total catchment population of this facility _________________

2.2 Expected pregnancy registration in this facility per month _____________________________

(it maybe number attended per 1000 population)

2.3 Does this facility have in patient facility? Yes ___________ No __________

2.4 How many births per month take place at this facility? ___________

2.5 Total number of in patients during the last year (2012) __________

2.6 Antenatal beds __________

2.7 Labour room beds _________________

2.8 Postnatal beds ________________

2.9 Total number of beds in the obstetric ward ____

2.10 Does this facility have an adult critical care unit? Yes _______ No ______

2.11 Does this facility have a NICU? Yes____________ No__________

SECTION 3: AVAILABILITY OF MATERNITY SERVICES

**Service**	**Availability # of times per week**	**24 hours**	**Day time only**	**Comments (if any)**
**Emergency Care**				
**Maternal**				
**Antenatal**<br>**Labor & Delivery**<br>**Postnatal**				
**Neonatal**				
**Outpatient Department**				
**Maternal**				
**Antenatal**<br>**Postnatal**				
**Neonatal**				

SECTION 4: MATERNITY SERVICES HUMAN RESOURCE NUMBERS

Staff	Total Number	Full time	Part-time
**Obstetricians**			
**Neonatologists**			
**Pediatricians**			
**Anesthesiologists**			
**Medical Officers**			
**Staff Nurses**			
**Radiologists**			
**Lab Technicians**			
**ANMS**			
**Other**			
**Other**			
**Other**			

SECTION 5: SUPPORT FOR HUMAN RESOURCES

	Yes	No
• If staff is to be called for an **obstetrical emergency**, does the facility provide transport?		
• If staff is to be called for a **neonatal emergency**, does the facility provide transport?		
• If yes, is transport available 24x7?		
• Has any of your staff received training for management for pregnant women with hypertension during or immediately after delivery?		
• Has any of your staff received training for management for pregnant women with hypertension and with fits during or immediately after delivery?		
• If No then do you believe that training for management of pregnant women with hypertension/and with fits would be beneficial?		
• If Yes, what type of training you think would be required?		

TRAINING

**Document training completed in the last 6 months**	**Yes**	**No**
• Was any training completed		
• If training, what type (please check all that apply):		
• Pre-Eclampsia<br>• Neonatal Resuscitation Programe		
• PPH		
• Prolonged labour		
• Sepsis		
• Immediate newborn care		
• Emergency Preparedness		
• C-Section		
• Post-abortion care		
• Site preparation		
• Essential newborn Care		
• Other, Specify ______		

SECTION 6: LAB SERVICES AVAILABLE TO THIS FACILITY

**Type of lab test**	**Available (Yes/no)**	**Wait time for results (hours)**	**Cost to patient (rupees)**
Platelets			
Creatinine			
AST/ALT			
PT/aPTT/INR			
Dip-stick for proteinuria			
Protein: creatinine ratio			
Fetal heart rate by CTG			
Fetal heart rate by Doppler			
Ultrasound for fetal growth			
Ultrasound for amniotic fluid volume			
Umbilical artery Doppler			

SECTION 7: MEDICATIONS FOR MANAGEMENT OF HYPERTENSION IN PREGNANCY/PRE-ECLAMPSIA/ECLAMPSIA

**Medications**	**Present in facility Yes/No**	**Available in labour room Yes/No**
Atenolol PO		
Labetalol IV		
Labetalol PO		
Nifedipine PO (Capsules/tablets)		
Nifedipine SL (Capsules/tablets)		
Methyldopa PO		
Hydralazine IV		
Hydralazine PO		
Other antihypertensive (specify) :		
Betamethasone IM		
Dexamethasone IM		
Heparin SC		
Heparin IV		
$MgSO_4$ (Specify concentration and g/amp)		
Calcium gluconate IV		
Calcium gluconate IM		
Diazepam IV		
Diazepam PO		
Oxytocic drugs (InjectionOxytocin, injection Ergometrine)	List available	List available in labour room

7.1 Does this facility ever experience stock-outs of MgSO4? If yes, how often in last six months?

____

____

____

____

SECTION 8: PROCEDURES AVAILABLE

**Maternal Care**	**Present in facility Yes/No/NA(Not Available)**	**Available in labour room**
BP Measuring apparatus		
Maternal weighing machine		
Assisted delivery by vacuum extraction		
Assisted delivery by forceps		
Manual removal of placenta		
Removal of retained products		
Blood transfusion (mention maximum units available to one patient)		
Cesarean section capacity 24x7		
If not available 7 days a week and 24 hours a day, please state availability		
Time to conduct emergency Cesarean section (Minutes ___________)		
Hysterectomy capacity		
**Newborn Care**		
Minimum gestational age for resuscitation		
Minimum gestational age for care at this facility		
Minimum birth weight for resuscitation		
Minimum birth weight for care at this facility		
Basic newborn resuscitation		
Clean cord care		
Phototherapy lights		
Incubator care		
Ambient O2		
CPAP care		
Ventilation support		

SECTION 9: TRANSPORT

Transport	Yes	No	Don't know
**Patient transport**<br>Does this facility provide ambulance service?			
If yes, is it available 24x7?			
If it is not available 24x7?, when is it available:<br>Mention timing			
What is the catchment area for the ambulance service?			
What are the ambulance charges to the patient?			

SECTION 10: FACILITY GUIDELINES FOR HYPERTENSIVE DISORDERS OF PREGNANCY

Resource for hypertensive disorders of pregnancy	Yes	No	Don't know
Is there a guideline for care of women with hypertensive disorders of pregnancy at this institution?			
Is this guideline available to all staff?			
Can interviewer have access to/copy of the guideline?			
Is there an institutional policy specifically for prevention of pre-eclampsia?			
Is there an institutional policy specifically for treatment of eclampsia?			
Are posters/charts displayed?			

11.1 How is severe hypertension in pregnancy defined at this institution in terms of systolic and diastolic BP? sBP _______________ dBP ___________________

11.2 Which antihypertensive drugs are used to control severe hypertension in pregnancy?

Name of drug _______________ Dose ____________ Route ______________

Name of drug _______________ Dose ____________ Route ______________

Name of drug _______________ Dose ____________ Route ______________

11.3 is magnesium sulphate used to manage patients with **severe pre-eclampsia**?

Yes _____________ No ______________

If yes, loading dose with route ___________________

Maintenance dose with route ___________________

Please list other anti-convulsant medications used in pre-eclampsia (name, dose, route, indication)

____
____
____

11.4 Is magnesium sulphate used to manage patients with **eclampsia?**

Yes __________________ No _______________

If yes, loading dose with route _______________

Maintenance dose with route ________________

Please list other anti-convulsant medications used ineclampsia (name, dose, route, indication)

____

____

SECTION 11: FACILITY MATERNAL-NEWBORN MORTALITY AND MORBIDITY DATA

12.1 How the facility collects data regarding mothers -newborns in their catchment area?

____

____

____

____

SECTION 12:EmOC

13.1 DOES this facility meet the UNFPA requirements for **Basic EmOC**?

To be performed **without the need of an operating theatre:**

IV/IM antibiotics	Yes _______	No________
IV/IM oxytocics	Yes _______	No________
IV/IM anticonvulsants	Yes _______	No________
Manual removal of placenta	Yes _______	No________
Assisted vaginal delivery	Yes _______	No________
Removal of retained products	Yes _______	No________

13.2 Does this facility meet the UNFPA requirements for **Comprehensive EmOC?**

To be performed **with an operating theatre:**

IV/IM antibiotics	Yes _______	No________
IV/IM oxytocics	Yes _______	No________
IV/IM anticonvulsants	Yes _______	No________
Manual removal of placenta	Yes _______	No________
Assisted vaginal delivery	Yes _______	No________
Removal of retained products	Yes _______	No________
Cesarean section	Yes _______	No________
Blood transfusion	Yes _______	No________

Please complete for this facility for the previous 12 months

**Birth**	
Total unassisted vaginal births	
Total assisted vaginal births (vacuum extraction & forceps)	
Total cesarean section births	
Total number of term deliveries >37 weeks	
Total number of preterm deliveries <37 weeks	
**Surgeries**	
Total manual removal of placenta	
Total hysterectomies	
**Referrals**	
Total number of maternity referrals to this facility	
Total number of referrals for hypertensive disorder of pregnancy and/or Eclampsia	
Number of referrals out of this facility	
**Maternal mortality**	
How many women who were pregnant or who had just delivered (within 42 days after delivery), died at this facility during last one year?	
**Maternal morbidities**	
Number of women with Pre-eclampsia	
Number of women with Eclampsia	
Number of women with antepartum hemorrhage	
Number of maternity patients with sepsis	
Number of women with prolonged or obstructed labour	
Number of maternity patients with stroke	
Number of women with miscarriage<br>Number of women with medical termination of pregnancy	
**Perinatal mortality**	
Number of stillbirths >28 weeks	
Number of neonatal deaths within 7 days of life	
Number of neonatal deaths 8-28 days of life	
**Neonatal morbidities (In numbers)**	
In term neonates > 37 weeks	
In preterm neonates <37 weeks	
